# Quantitative analysis of peripapillary capillary volume using dense B-scan OCT angiography in normal and diabetic retina

**DOI:** 10.1186/s40662-024-00402-4

**Published:** 2024-09-01

**Authors:** Lingyan Zeng, Xin Liu, Shuyu Chen, Jin Ma

**Affiliations:** State Key Laboratory of Ophthalmology, Zhongshan Ophthalmic Center, Sun Yat-Sen University, 7# Jinsui Road, Guangzhou, 510000 China

**Keywords:** Dense B-scan optical coherence tomography angiography, Peripapillary capillary volume, Diabetic retinopathy

## Abstract

**Background:**

The value of quantitatively analyzing peripapillary capillary volume (PPCV) distribution was explored in normal and diabetic retinopathy (DR) eyes using dense B-scan optical coherence tomography angiography (DB OCTA).

**Methods:**

This was a cross-sectional observational study followed by prospective follow-up for those with DR, which enrolled 101 healthy subjects and 140 DR patients. Dense, automatic, real-time (DART) volume scans of DB OCTA were performed using a Spectralis HRA + OCT2. ImageJ and MATLAB were used to process and calculate PPCV distribution detected by DB OCTA.

**Results:**

In normal subjects, PPCV distribution were significantly correlated with the age and quadrant location (all *P* < 0.001). The PPCV distribution in each quadrant was significantly lower in severe nonproliferative DR patients than in normal subjects in all age groups (all *P* < 0.05, t-test). Compared to normal subjects, the PPCV distribution improved significantly in the pan-retinal photocoagulation treatment and surgery groups (all *P* < 0.001). No significant variation was observed in the anti-VEGF treatment group and normal subjects (*P* > 0.05). The PPCV distribution is significantly correlated with post-treatment best-corrected visual acuity in both the pan-retinal photocoagulation treatment and surgery groups (all *P* < 0.003) but not in the anti-VEGF treatment group (*P* = 0.940).

**Conclusions:**

Quantitative assessment of PPCV distribution using DB OCTA is valuable in prognosis evaluation of DR with pan-retinal photocoagulation and surgery.

## Background

Researchers [1, 2] have explored the distribution of peripapillary capillary density in each superficial and deep retinal layer using conventional optical coherence tomography angiography (OCTA) *en face* (C-scan) images. The results show that peripapillary microcirculation has changed in the early stage of diabetic retinopathy (DR), and conventional OCTA can quantitatively investigate the microvascular density of each retinal layer [3]. However, due to the limitations of conventional OCTA, it is unknown whether the three-dimensional (3D) volume of microcirculation has changed.

Recently, a novel approach to volume-rendering OCTA has been used to represent 3D retinal vascular models and acquire in-depth capillary blood flow information [4–7]. This spatial volume-rendering technique calculates the geometric flow volume of the retinal microcirculation by integrating structural information from the reflectance optical coherence tomography (OCT) with flow information derived from *en face* OCTA. However, some studies suggest that retinal capillary plexuses are arranged more parallelly than vertically [8, 9], indicating that partial parallel capillary flow information could be lost when using *en face* OCTA images to reconstruct 3D views of retinal microcirculation volume. However, OCTA B-scan images contain cross-sectional signals of full-thickness parallel retinal capillary flow, which could reduce signal loss in capillary flow that runs in parallel between C-scan layers. Moreover, retinal layer segmentation errors can confound the interpretation of *en face* OCTA in diseases causing distorted retinal structures [4]. Additionally, blood flow projected from more superficial layers (projection tails) can further confuse the interpretation of *en face* views [10, 11]. Therefore, using a B-scan OCTA-based 3D capillary volume reconstruction model may be more precise and objective. However, the low quality of flow signal caused by the low number (2–7 scans) of repeated B-scans in current commercial devices makes it difficult to reconstruct accurate 3D retinal vascular model. With the evolution of OCTA technology, Freund et al. [12] described the method of dense B-scan (DB) OCTA that enables the evaluation of retinal capillary blood flow at a higher resolution than conventional acquisitions. Thin, dense raster scans are used to produce highly resolved structural B-scans with superimposed flow signals that provide precise correlations between the retinal microstructure and blood flow. At each tissue location, 12–100 repeated OCT B-scans are used by the full-spectrum probabilistic OCTA algorithm to determine the presence or absence of flow at each voxel. In DB OCTA, a scan model of dense, automatic, real-time (DART) volume consisting of 25 high-quality B-scans with an interscan distance of 6 μm nominal spacing could continually provide 25 cross-sectional areas of blood flow in the region of interest (10° × 0.5°).

Thus, we utilized the DART volume scan mode to reconstruct retinal capillary volume with the assistance of ImageJ (v. 1.8.0, NIH, USA) and MATLAB (v. 9.1.0, MathWorks Company) software in this study. Peripapillary capillary volume (PPCV) distribution were analyzed in normal and severe nonproliferative DR eyes. In severe nonproliferative DR eyes, it was compared before and after the following treatment with intravitreal anti-vascular endothelial growth factor (anti-VEGF) injections, pan-retinal photocoagulation (PRP), or 27-gauge (G) vitrectomy.

## Methods

### Subjects

In this cross-sectional observational study followed by prospective follow-up for those with DR, we enrolled 101 healthy subjects (101 eyes) and 140 patients (187 eyes) with DR (secondary to type 2 diabetes). For healthy participants, the eye was chosen at random for inclusion in the study analysis, and the inclusion criteria were a best-corrected visual acuity (BCVA) of 20/20 or better and refraction less than or equal to − 3 D. Subjects with a family history of glaucoma in a first-degree relative; signs of myopic degeneration, a pathological form of myopia, or other ophthalmic diseases; or the presence of any systemic disease that may affect retinal blood flow, such as hyperlipidemia nephropathy or hypertension, were not included in the analysis. DR was diagnosed and graded according to the International Clinical Diabetic Retinopathy Disease Severity Scales [13]. Eyes with either proliferative DR or severe nonproliferative DR but with no evidence of other ocular disorders (e.g., optic neuropathies, retinal vein occlusion, age-related macular degeneration, glaucoma, myopic maculopathy, and epiretinal membrane) were included in the study. Patients with poor media clarity due to significant cataract or vitreous hemorrhage were excluded from the study. Out of 98 patients with severe nonproliferative DR, 136 eyes were enrolled. Eyes with a history of PRP treatment or intravitreal anti-VEGF injections within the previous 6 months were excluded. The enrolled eyes that required PRP treatment were 74 eyes (PRP treatment group) or intravitreal anti-VEGF injections of Aflibercept (Eylea, Bayer AG, Leverkusen, Germany) were 62 eyes (anti-VEGF treatment group). Out of 42 patients with proliferative DR, 51 eyes were enrolled. All of these eyes with tractional retinal detachment or tractional macular edema required 27-G vitrectomy and endolaser PRP treatment (surgery treatment group). Cases with tractional retinal detachment or neovascularization within three papilla disks (PD) of the edge of the optic disk were excluded. The enrolled DR patients came for clinical follow-up at 1 and 2 weeks and 1, 2, and 3 months post-treatment, the observation indices included ophthalmic examination, BCVA and DB OCTA. All subjects were enrolled at Zhongshan Ophthalmic Center, Sun Yat-sen University, from March 2021 to December 2022. All patients were diagnosed and operated on by the same experienced ophthalmologist (JM). The research protocol was approved by the institutional ethics committee of Zhongshan Ophthalmic Center, Sun Yat-sen University (2023KYPJ218). All procedures that involved human participants adhered to the ethical standards of the institutional research committee, the 1964 Declaration of Helsinki, and its later amendments or comparable ethical standards. Informed consent was obtained from all participants included in the study.

### DB OCTA data acquisition, processing and PPCV evaluation

**Fig. 1:**
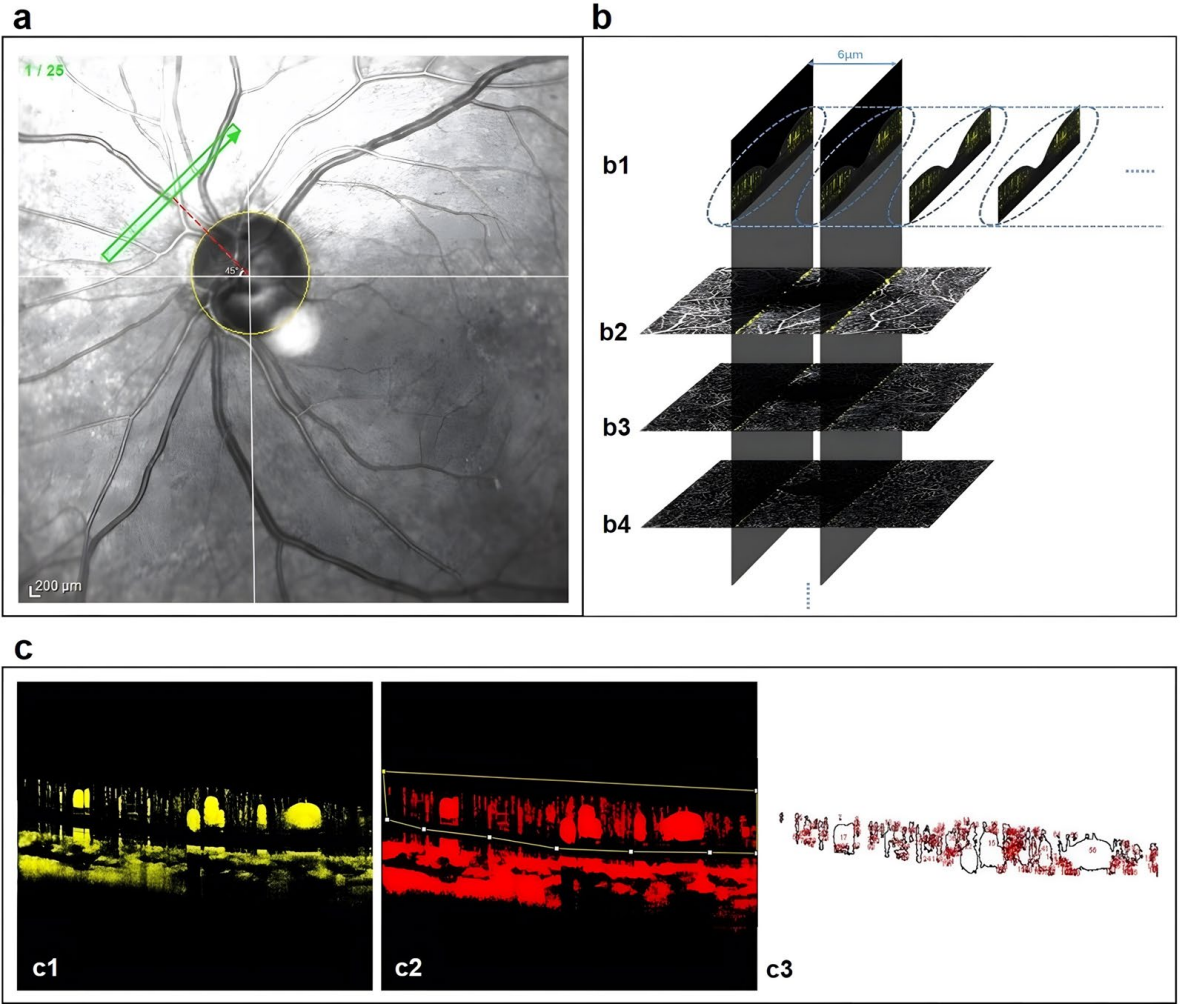
Graphical representation for PPCV reconstruction using DB OCTA. **a** Representative image obtained from a DB OCTA examination. Each peripapillary area was subdivided into four regions of interest—the temporal superior quadrant, temporal inferior quadrant, nasal superior quadrant, and nasal inferior quadrant. The scan area was 10° × 0.5° (3 mm × 0.15 mm), and the scan angle was 45° for all subjects. The scan was moved manually at 1/2 PD of the edge of the optic disk. **b** High-quality B-scan OCTA images acquired using the dart-volume scan mode of DB OCTA for retinal capillary flow volume reconstruction. **b1** All 25 high-quality B-scan OCTA images from one area of interest with an interscan distance of 6-μm in nominal spacing were used for retinal blood flow volume reconstruction. Each B-scan OCTA image contained all retinal blood vessels within the same cross-section, including the superficial **(b2)**, intermediate **(b3)**, and deep **(b4)** vascular plexus. **c** A high-quality B-scan OCTA image loaded into ImageJ software to analyze the retinal cross-sectional capillary flow area. **c1** The signal graphics in yellow represent blood flow detected by DB OCTA. **c2** ImageJ was used to adjust the brightness and threshold of the images and depict the blood flow signals that fell within the threshold setting as red. The “polygon selections” tool was used to define the region of retinal blood flow (the area within the yellow lines). **c3** The area of the retinal blood flow signal was determined by selecting “analyze > measure” command in ImageJ. Blood vessel areas (the areas confirmed by the expert ophthalmologist) were excluded to calculate the retinal capillary flow area. PPCV, peripapillary capillary volume; DB OCTA, dense B-scan optical coherence tomography angiography

**Fig. 2:**
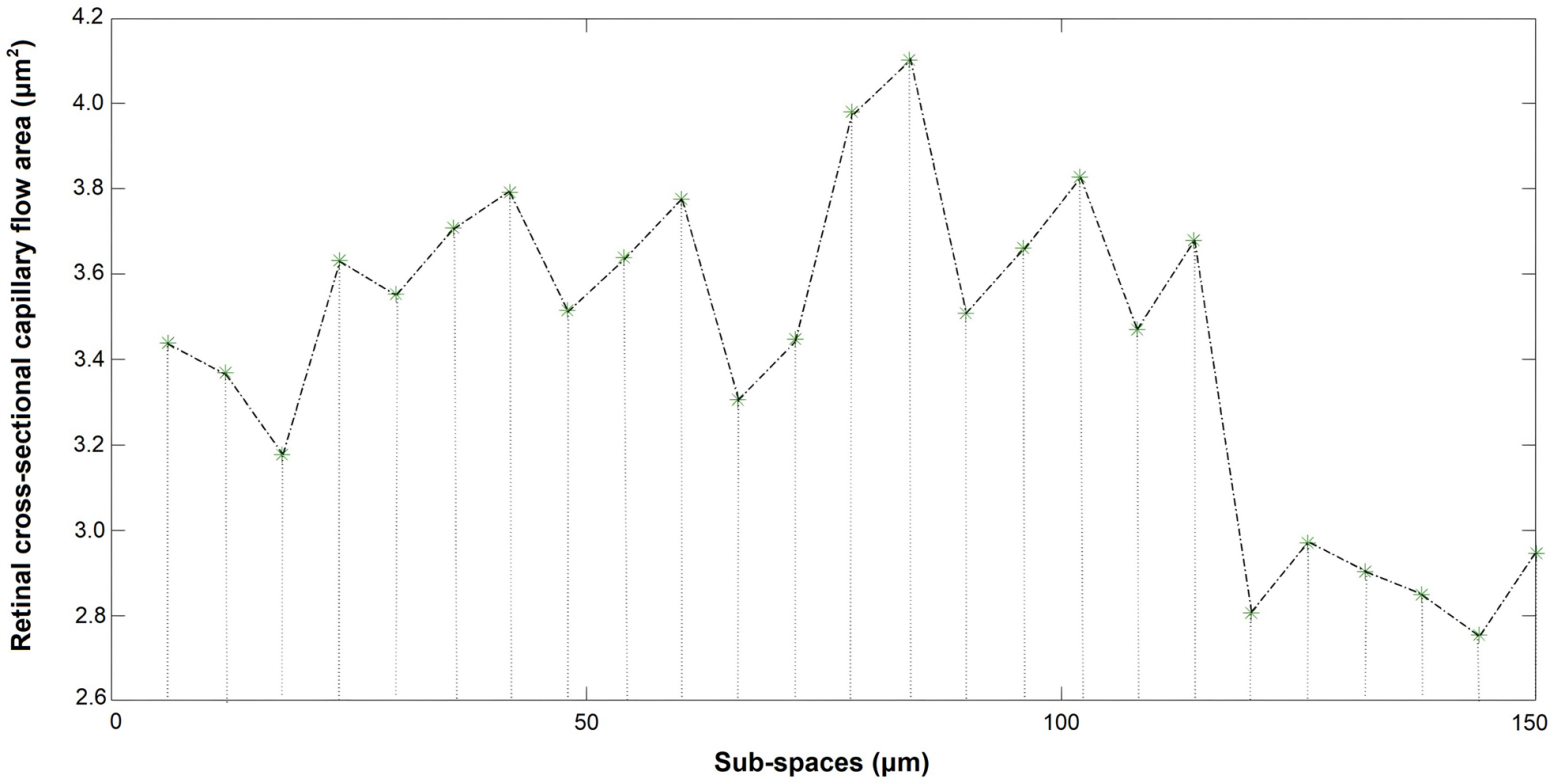
PPCV was calculated using the trapezoidal rule integration method. The segment of retinal microcirculation scanned by DB OCTA was divided into several sub-spaces, with a slice interval of 6 μm. Retinal microcirculation volume in each sub-space was calculated approximately by MATLAB software. PPCV, peripapillary capillary volume; DB OCTA, dense B-scan optical coherence tomography angiography

DB OCTA acquisitions were acquired after pupil dilation as described previously [12]. All eyes underwent DB OCTA examination using manufacturer's software applied to Heidelberg Spectralis OCTA scans: Heidelberg SP-X2001 (Heidelberg Engineering, Heidelberg, Germany), with a central wavelength of 870 nm and an 85 kHz A-scan rate. A DART volume scan mode was performed in this study. These scans were performed 1/2 PD to the side of the optic disk and at a 45-degree angle to the horizontal line in four peripapillary quadrants (temporal superior, temporal inferior, nasal superior, and nasal inferior), which were clearly marked by an auxiliary external crosshair manually centered on the optic nerve head (Fig. 1a). DART volume scan pattern covered a 10° × 0.5° (~ 3 × 0.15 mm) area and comprised 25 B-scans (512 A-scans each) with an approximately 6-μm spacing between each. This is equal to 25 × 3 × 4 up to 25 × 25 × 4 B-scan repeats, or 300–2,500 OCT B-scans in total, per DART volume scan (Fig. 1b). The images obtained were processed with ImageJ software and MATLAB software for analysis. Subjects with poor-quality DB OCTA images (e.g., due to eye movement) were not included in the analysis. ImageJ software was used to process and calculate the retinal cross-sectional capillary flow area detected by DB OCTA as well as to adjust the brightness and threshold of each image (Fig. 1c). The same brightness and threshold values were set in each picture. All images were independently analyzed and compared among healthy subjects and DR patients by two experienced ophthalmologists. The retinal cross-sectional capillary flow areas measured were input into MATLAB software, and the PPCV was calculated using the trapezoidal rule integration method. The retinal cross-sectional capillary flow areas measured were input into MATLAB software (Fig. 2). The MATLAB language (MathWorks Company) was used for the numerical integration of PPVC in this study. The basic principle was to divide the integral space (a 3 mm × 0.15 mm segment of retinal microcirculation scanned by DB OCTA) [a, b] into several sub-spaces (6-μm interscan spacing); x_i_, x_i+1_, i = 1, 2, … n, where x_1_ = a, x_n+1_ = b. The division of the integral space is shown in the following formula:

$$\begin{gathered} {\text{V}} = \,\int_{{\text{a}}}^{{\text{b}}} {{\text{A}}\left( {\text{x}} \right){\text{ dx}}} \hfill \\ \quad = 1/2\,\left[ {{\text{A}}\left( {{\text{x}}_{1} } \right) + {\text{A}}\,\left( {{\text{x}}_{{{\text{n}} + 1}} } \right)} \right]\,\Delta {\text{h}}\, + \,\sum\nolimits_{{{\text{i}}\, = \,2}}^{{{\text{n}}\, - \,1}} {\left[ {{\text{A}}\,\left( {{\text{x}}_{{\text{i}}} } \right) + {\text{A}}\,\left( {{\text{x}}_{{{\text{i}} + 1}} } \right)} \right]\Delta {\text{h}}} \hfill \\ \end{gathered}$$

### Statistical analysis

All statistical analysis was performed with Statistical Package for the Social Sciences (SPSS, version 18.0, Chicago, IL), and all data were described as mean ± standard deviation (SD). A *P* value less than 0.05 was considered statistically significant. The Student–Newman–Keuls (SNK) test and the least-significant difference (LSD) test was used for pairwise multiple comparisons of PPCV distribution. The statistically significant differences in the means of PPCV distribution between the severe nonproliferative DR group and the control group was evaluated using Student's t-test. We used generalized estimating equations (GEE) analysis to evaluate the possible intereye correlation. A simple linear regression analysis and a multiple linear regression analysis were performed to analyze the factors influencing PPCV distribution. All results were compared between ophthalmologists using the intraclass correlation coefficient (ICC). The analysis from one ophthalmologist was repeated twice to calculate repeatability (κ). Results from one ophthalmologist were reported in this study as the mean and standard deviation for all eyes.

## Results

PPCV distribution showed high reproducibility between ophthalmologists with an ICC of 0.937. The analysis from one ophthalmologist was repeated twice to determine repeatability (κ = 0.986).

### Patient characteristics

The study included 101 healthy subjects and 140 type 2 diabetic mellitus patients with DR. The demographics and baseline characteristics of the two groups are shown in Table 1.

**Table 1:** Demographics of healthy and diabetic subjects

Parameter	Healthy (n = 101)	DR (n = 140)	*P* value
Age (years)	46.79 ± 19.30	58.34 ± 15.78	< 0.001*
Male/Female	50/51	74 / 66	0.721
HbA1c (%)	–	6.82 ± 1.15	–
Diabetes mellitus duration (years)	–	11.56 ± 3.95	–
SBP (mmHg)	125 ± 8	134 ± 7	0.213
DBP (mmHg)	75 ± 9	83 ± 6	0.091
IOP (mmHg)	15.25 ± 1.78	15.33 ± 2.04	0.771
BMI (kg/m^2^)	22.37 ± 0.92	22.42 ± 1.28	0.875

*DR* = diabetic retinopathy; *SBP* = systolic blood pressure; *DBP* = diastolic blood pressure; *IOP* = intraocular pressure; *BMI* = body mass index; *P < 0.05

### Distribution of PPCV in normal subjects

**Fig. 3:**
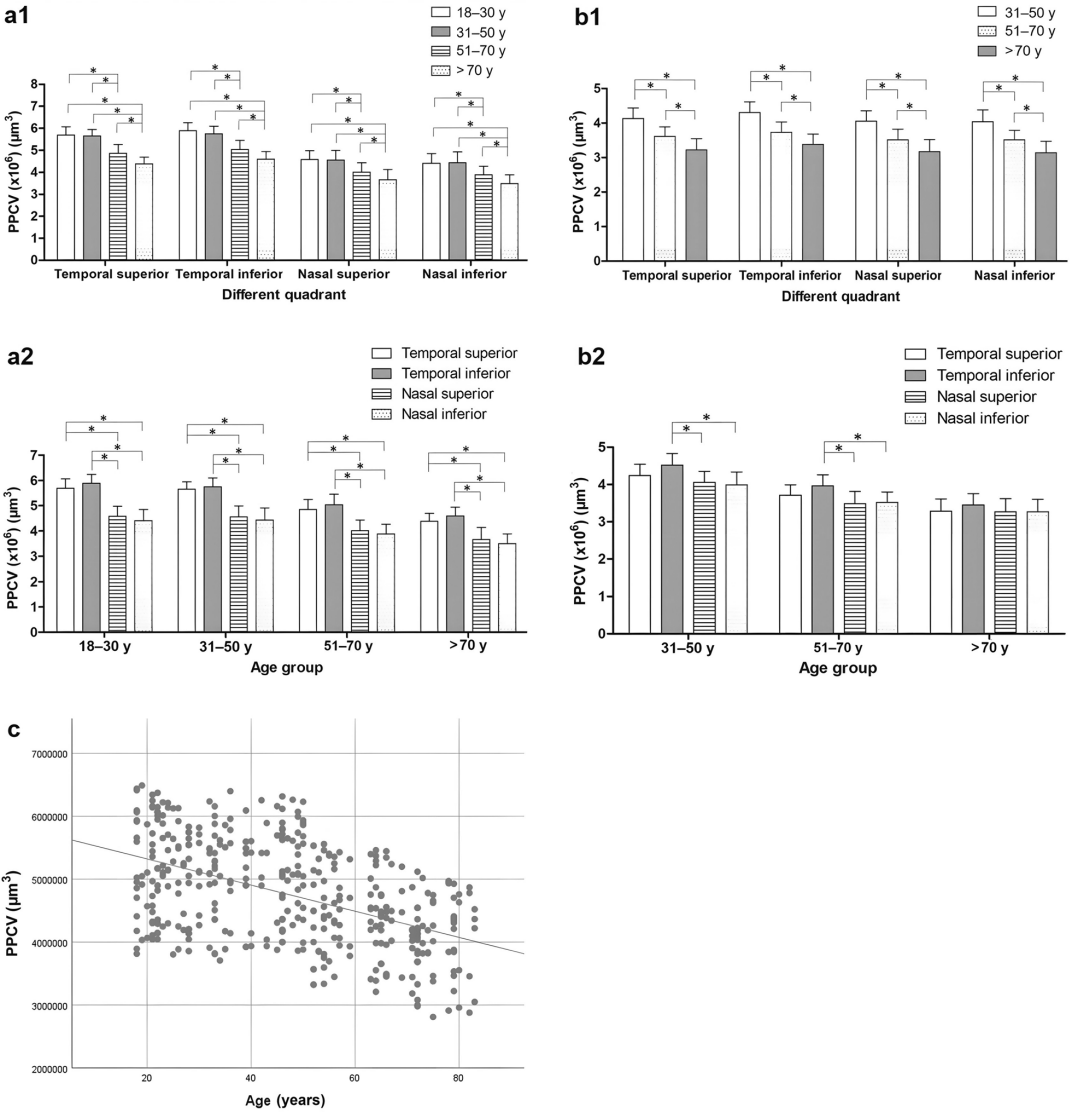
Distribution of PPCV in normal subjects and severe nonproliferative DR cases. Comparison of PPCV distribution in normal subjects among different age groups **(a1)** and quadrants **(a2)**, and in severe nonproliferative DR cases among different age groups **(b1)** and quadrants **(b2)**. *P < 0.05 (SNK test). **a1** There was no significant difference in PPCV distribution between the 18–30 and 31–50 years age groups in any of the four quadrants (all *P* > 0.05, SNK test). **a2** There were no significant PPCV distribution differences between the temporal superior and inferior quadrants or between the nasal superior and inferior quadrants in any age group (all *P* > 0.05, SNK test). **b2** There were no significant differences in PPCV distribution between any other quadrants in the 31–50 and 51–70 age groups (all *P* > 0.05, SNK test), and there were no significant differences in PPCV distribution between the four quadrants in subjects over 70 years (all *P* < 0.05, SNK test). **c** Scatterplot of PPCV distribution vs. age in normal subjects showing a best-fit simple regression line. The overall fit is significant (*P* < 0.001). PPCV, peripapillary capillary volume; DR, diabetic retinopathy; y, years

The subjects were divided into four age groups (18–30, 31–50, 51–70, and > 70 years old; Fig. 3a1, a2). When the PPCV distribution was compared among different quadrants in the same age group (Fig. 3a1), the distributions in the temporal inferior and superior quadrants were significantly higher than those at the nasal superior and inferior quadrants in all four age groups (all *P* < 0.05, SNK test). In addition, when PPCV distribution was compared among different age groups in the same quadrant (Fig. 3a1), PPCV distribution in the over-70 age group was significantly lower than that in any of the other three age groups, and the 51–70 age group’s value was significantly lower than those of the 18–30 and 31–50 age groups (all *P* < 0.05, SNK test).

### Multiple linear regression analysis of factors concerning PPCV distribution in normal subjects

The regression variables were age, gender, and quadrant location of PPCV (temporal and nasal hemisphere). Gender and quadrant location of PPCV were regarded as binary variables. Since, as mentioned above, there was no significant difference in PPCV distribution in any age groups between the superior and inferior quadrants in either the temporal or nasal region, PPCV distributions in the temporal superior-inferior quadrants were regarded as variables of quadrant locations of PPCV at the temporal hemisphere, while PPCV distributions in the nasal superior-inferior quadrants were regarded as variables of quadrant locations of PPCV at the nasal hemisphere. The variance inflation factors (VIF) for gender, age, and quadrant location of PPCV were all 1.000, and no evidence of multicollinearity was found. A scatter plot (Fig. 3c) showed a linear correlation between PPCV distribution and age. In multiple regression analysis, age and quadrant location of PPCV were shown to be significantly correlated with PPCV distribution (β =  − 20790.03, and β =  − 1.129 × 10^6^, respectively, all *P* < 0.001). However, the effect of gender (*P* = 0.855) was negligible from a statistical point of view.

### Distribution of PPCV in severe nonproliferative DR cases

**Fig. 4:**
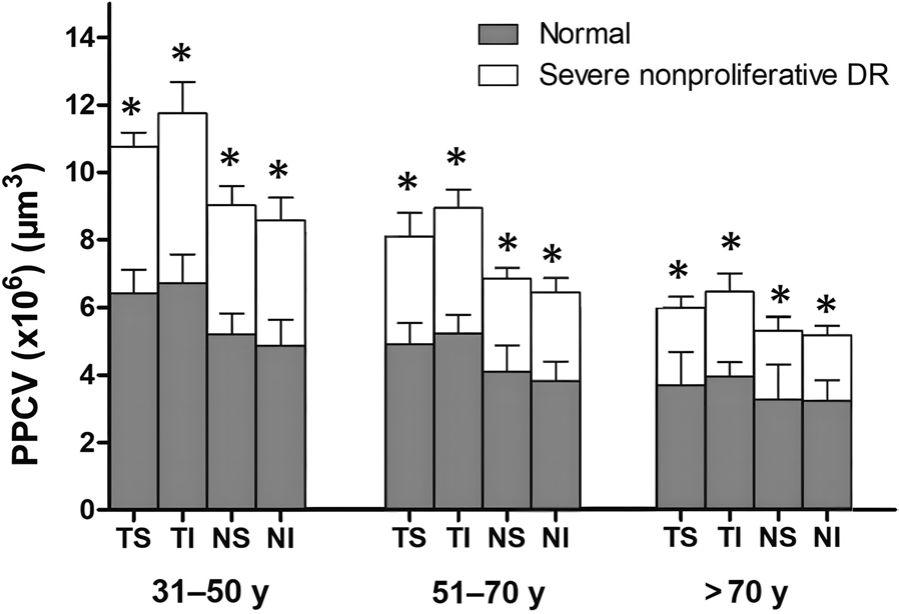
Comparison of PPCV distribution between severe nonproliferative DR cases and normal subjects. Groups were compared using generalized estimating equation to correct for intereye associations. PPCV, peripapillary capillary volume; DR, diabetic retinopathy; TS, temporal superior; TI, temporal inferior; NS, nasal superior; NI, nasal inferior; y, years; *P < 0.05 (t-test)

In consideration of the small number of youth (younger than 30 years) with type 2 diabetes, the subjects were divided into three age groups (31–50, 51–70, > 70 years) (Figs. 2b2 and 3b1). When the PPCV distribution was compared among different quadrants in the same age group (Fig. 3b2), the PPCV distribution at the temporal inferior quadrant was significantly higher than those at the nasal superior and nasal inferior quadrants (all *P* < 0.05, SNK test). In addition, when PPCV distribution was compared among different age groups in the same quadrant (Fig. 3b1), it was significantly higher in the 31–50 age group than in the other two age groups. In the over-70 age group, it was significantly lower than in the 51–70 age group in all four quadrants (all *P* < 0.05, SNK test). PPCV distributions were compared between severe nonproliferative DR cases and normal subjects (Fig. 4). In all age groups, the PPCV distribution in each quadrant was significantly lower in severe nonproliferative DR patients than in normal subjects (all *P* < 0.05, t-test).

### Changes in PPCV distribution in DR patients before and after treatment

**Fig. 5:**
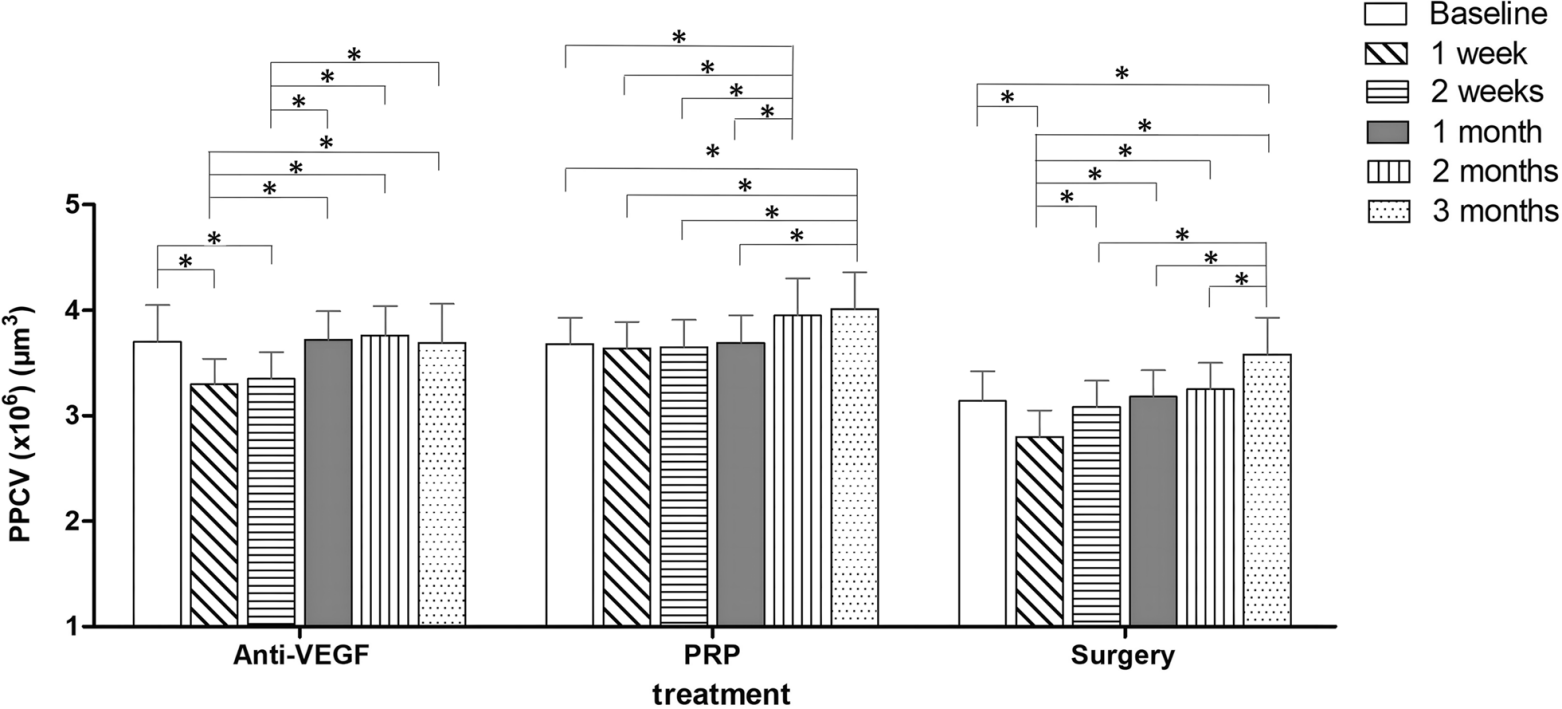
Post-treatment change in PPCV distribution in DR patients. Patients came for clinical follow-up at 1 and 2 weeks and 1, 2, and 3 months post-treatment. *P < 0.05 (LSD test and SNK test). PRP, pan-retinal photocoagulation; DR, diabetic retinopathy; PPCV, peripapillary capillary volume

The change in PPCV distribution over time after DR treatment is illustrated in Fig. 5. In the anti-VEGF treatment group, PPCV distribution at weeks 1 and 2 was significantly reduced compared with baseline (all *P* < 0.001, LSD test). Pairwise comparison among the post-treatment groups showed that PPCV distribution at weeks 1 and 2 were significantly lower than that at months 1, 2, and 3 (all *P* < 0.05, SNK test). In the PRP treatment group, PPCV distribution at months 2 and 3 was significantly increased compared with baseline (all *P* < 0.001, LSD test). Pairwise comparison among the post-treatment groups showed that PPCV distribution at weeks 1 and 2 and month 1 were significantly lower than at months 2 and 3 (all *P* < 0.05, SNK test). In the surgery treatment group, the PPCV distribution at week 1 was significantly lower than baseline (*P* < 0.001, LSD test), and at month 3, it was significantly higher than baseline (*P* < 0.001, LSD test). Pairwise comparison post-treatment showed that the PPCV distribution at week 1 was significantly lower than that at week 2 and at months 1, 2, and 3 (all *P* < 0.05, SNK test), while at month 3, it was significantly higher than at week 2 and months 1 and 2 (all *P* < 0.05, SNK test).

**Fig. 6:**
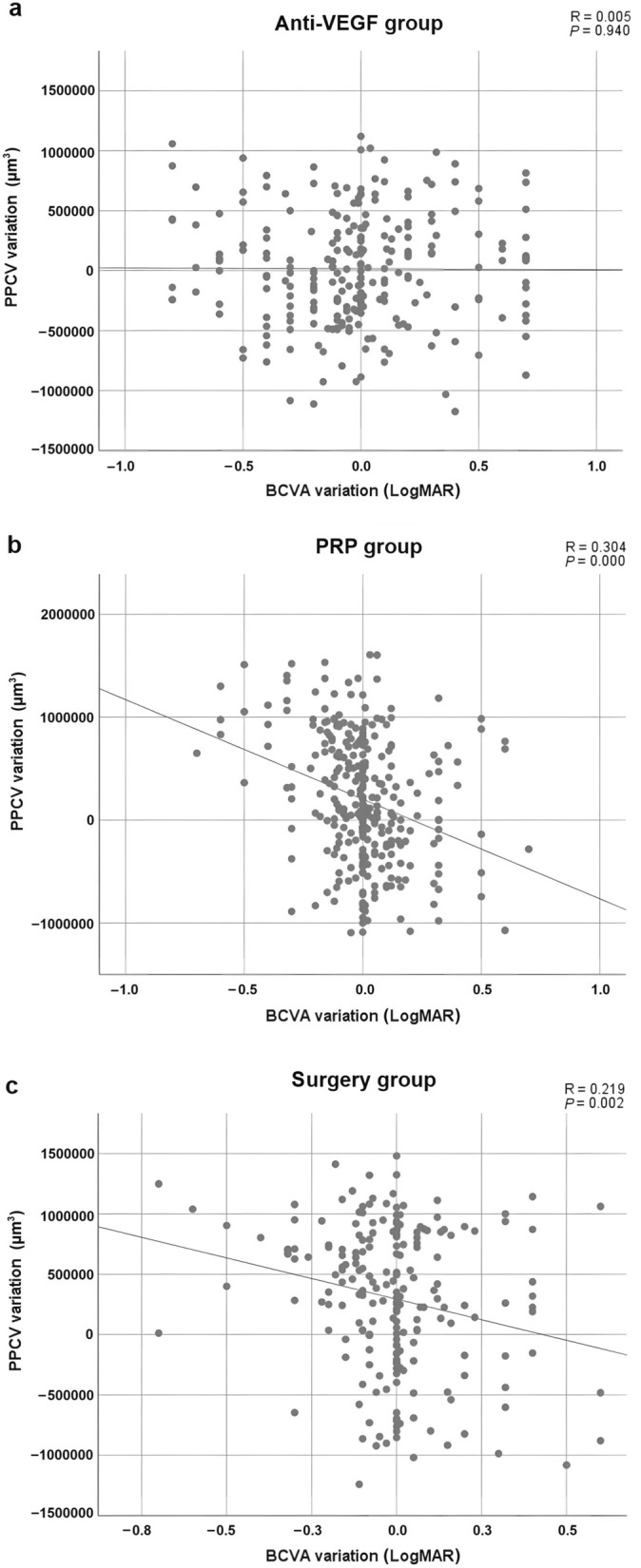
Scatterplots and linear regression analyses of variation in PPCV vs. variation in LogMAR BCVA before and 3 months after anti-VEGF (**a**), PRP (**b**), or surgery (**c**). BCVA, best-corrected visual acuity; PPCV, peripapillary capillary volume; PRP, pan-retinal photocoagulation; anti-VEGF, anti-vascular endothelial growth factor

In the anti-VEGF treatment group (Fig. 6a), no correlation was observed in the variation in PPCV distribution and the variation in LogMAR BCVA before and 3 months after treatment (*P* = 0.940). A scatter plot (Fig. 6b, c) showed that the variation in PPCV distribution was negatively and significantly correlated with the variation in LogMAR BCVA before and 3 months after PRP or surgery (β =  − 9.67 × 10^5^, and β =  − 6.83 × 10^5^, respectively, all *P* < 0.003).

## Discussion

Several researchers have used traditional OCTA to demonstrate peripapillary retinal perfusion in normal subjects [2, 14–17]. However, they studied the peripapillary microcirculation by measuring the surface and deeper layers of retinal microvascularization using the C-scan mode of traditional OCTA rather than assessing a general 3D view of peripapillary microcirculation, which better reflects peripapillary retinal perfusion status. To our knowledge, this is the first study presenting a method of volumetrically assessing peripapillary retinal microcirculation using DB OCTA. The results in this study indicate that DB OCTA is a valuable tool in PPCV measurement and thus improves its clinical utility. Our results showed that age and quadrant location of PPCV and DR were correlated significantly with PPCV distribution. PPCV distribution was greater in the temporal superior and inferior quadrants and decreases gradually after age of 50 years in normal subjects. Meanwhile, the age and quadrant location of PPCV both had significant effects on PPCV distribution in severe nonproliferative DR patients. PPCV distribution decreased significantly after 50 years in all four quadrants (temporal superior, temporal inferior, nasal superior, and nasal inferior), with a greater PPCV distribution decrease over time in severe nonproliferative DR. PPCV distribution was its greatest at the temporal inferior quadrant before 70 years of age, and were same among the four quadrants in patients over 70 years with severe nonproliferative DR. After matching for observed confounders (age and quadrant location of PPCV), our current study found that severe nonproliferative DR may cause a significant decrease in PPCV distribution in all quadrants and ages.

Among the findings in the model of retinal microvascularization, a predominantly parallel retinal microvascular network has been suggested [8, 9]. Some studies have reconstructed the volume of parimacular microcirculation by using traditional OCTA C-scan images [18, 19]. However, using the C-scan modes of traditional OCTA might result in some signal loss of capillary flow, which runs in parallel between the C-scan layers. In contrast, OCTA B-scans displays cross-section signals of full-thickness retinal capillary flow, which contain whole parallel layer signals of cross-sectional capillary plexuses. Therefore, it is more accurate to reconstruct a 3D view of retinal microcirculation volume using the B-scan mode of OCTA. Typical commercial instruments use OCTA acquisition protocols with two to seven repeated B-scans. Since they are acquired with minimal averaging, traditional OCTA B-scan images typically lack capillary flow details. These low-quality images make it impossible to reconstruct an accurate 3D view of retinal capillary volume. However, as a novel imaging technique, DB OCTA can use protocols with more repeated B-scans, which improves the quality of the final image and offers a more enhanced cross-sectional view of capillary flow. Hence, DB OCTA enables the evaluation of retinal capillary blood flow at higher resolution compared with conventional acquisitions [12].

In this study, PPCV distribution in the anti-VEGF treatment group showed transient but significant decreases at one and two weeks after treatment, suggesting that anti-VEGF treatment has a transient inhibitory effect on PPCV distribution which gradually diminishes over time. PPCV distribution in the PRP treatment group did not change 1 month after treatment but increased significantly at 2 and 3 months. This indicates that PPCV distribution may remain unchanged for a time before gradually increasing after PRP treatment. Mirshahi et al. also reported that eyes with severe nonproliferative DR show an increase in mean choroidal, luminal, and stromal areas at 6 months following PRP [20]. Furthermore, Faghihi et al. reported that the foveal avascular zone (FAZ) area became more circular and regular after PRP perhaps due to the reflow of the occluded capillary plexus [21]. In the surgery group, PPCV distribution decreased significantly at 1 week after treatment, gradually recovered from 2 weeks to 2 months, and increased significantly at 3 months after treatment. This is because the DR of the cases in the surgery treatment group was more severe than in the PRP treatment group, which caused a slow increase in PPCV distribution in the surgery group. In addition, membrane peeling and intraoperative PRP could lead to retinal injury and retinal edema, resulting in a significant decrease in PPCV distribution 1 week after surgery.

We found a statistically significant improvement in PPCV distribution after 3 months of treatment with PRP or surgery in DR patients. However, PPCV distribution did not significantly change after 3 months of intravitreal anti-VEGF injection treatment. This is because PRP and vitrectomy treatment could elicit a decrease in retinal ischemia and a reorganization of retinal blood distribution; the closure of the peripheral neovascular and hypoperfusion areas caused by PRP and vitrectomy might lead to an overall redistribution of retinal perfusion to the posterior pole and increased PPCV [21]. However, although anti-VEGF is believed to decrease angiogenic drive and regress neovascularization, it did not cause a reorganization of retinal microcirculation distribution. This may be why the key limitation of current anti-VEGF therapies in clinical use is their short-lived effects. Symptoms frequently recur as soon as the effect of an intravitreal injection wears off. This leads to the need for frequent anti-VEGF retreatments [22]. Otherwise, several studies [23–25] have shown that anti-VEGF treatment may cause the closure of normal retinal capillaries and a decrease in retinal microcirculation. Accordingly, the anti-VEGF treatment group did not show a significant increase in PPCV distribution after treatment as the PRP and surgery groups did. Similarly, in these three treatment groups, only the anti-VEGF treatment group showed no significant correlation between variation in PPCV distribution and BCVA after treatment. This is also because anti-VEGF therapy improves visual acuity mainly by promoting the resolution of macular edema rather than changing PPCV distribution, which is associated with the prognosis of the disease. The increase in PPCV distribution after treatment was significantly correlated with the improvement in BCVA in the PRP and surgery groups, providing a new objective indicator to evaluate the prognosis of visual function after PRP treatment and surgery. Thus, PPCV distribution increase is an important prediction signal for effective DR therapy.

There are several limitations to our study. First, the short follow-up in each treatment group might affect the results, which prevents us from making a firm conclusion regarding the association between alterations of PPCV and treatments. Second, the changes in the HbA1c and glycemic control of DR patients were not addressed in our study. However, significant changes are unlikely to happen in the relatively short-term follow-up period. Third, while the current study showed changes in the PPCV of diabetic patients following anti-VEGF, PRP, or surgery, the clinical relevance of these findings needs further investigations.

## Conclusions

In conclusion, PPCV distribution was significantly correlated with the age and quadrant location, which also improved significantly after 3 months of treatment with PRP or surgery in DR patients and correlated significantly with post-treatment BCVA in both the PRP treatment and surgery groups. Quantitative assessment of PPCV distribution using DB OCTA may be valuable in prognostic evaluation of DR with PRP and surgery.

## Data Availability

The data that support the findings of this study are available upon reasonable request from the corresponding author.

## Abbreviations

PPCV: Peripapillary capillary volume
DR: Diabetic retinopathy
DB OCTA: Dense B-scan optical coherence tomography angiography
DART: Dense, automatic, real-time
PRP: Pan-retinal photocoagulation
3D: Three-dimensional
anti-VEGF: Anti-vascular endothelial growth factor
BCVA: Best-corrected visual acuity
PD: Papilla disks
